# Ear Infection and Its Associated Risk Factors, Comorbidity, and Health Service Use in Australian Children

**DOI:** 10.1155/2013/963132

**Published:** 2013-06-06

**Authors:** Vasoontara Yiengprugsawan, Anthony Hogan

**Affiliations:** ^1^The Australian National University, National Centre for Epidemiology and Population Health Acton, Canberra, ACT 2601, Australia; ^2^ANZSOG Institute for Governance, University of Canberra, Acton, Canberra, ACT 2617, Australia

## Abstract

This study investigates and identifies risk factors, comorbidity, and health service use related to ear infection in Australian children. Two cross-sectional analyses of the Longitudinal Study of Australian Children (LSAC) involved 4,983 children aged 4 to 5 years in 2004 and aged 10 to 11 years in 2010. Odds ratios (ORs) were analysed using bivariate logistic regression. The prevalence of parent-reported ear infection was 7.9% (394) among children aged 4 to 5 years and 3.3% (139) at 10 to 11 years. Our study found that risk factors associated with ear infection were indigenous status, not being breastfed, mother or father smoking at least once a day, and father's school completion at year 9 or lower. By age 10 to 11 years significantly reported comorbidities were tonsillitis (OR 4.67; *P* < 0.001), headache (OR 2.13; *P* = 0.006), and asthma (OR 1.67; *P* = 0.003) and ear infection was found to be associated with the use of pediatrician (OR 1.83; *P* = 0.031), other specialist (OR 2.12; *P* < 0.001), and early intervention services (OR 3.08; *P* = 0.010). This empirical evidence can be used to inform the development of intervention and management programs for ear infection.

## 1. Introduction

Middle ear infection is a common, yet treatable disease and is a major cause of morbidity in children. If left untreated, long-term consequences of persistent severe ear infection can arise including speech development disorders [1], academic and educational development [2, 3], and lower overall quality of life [4, 5]. Subsequent hearing loss is one of the long-term implications of ear infection found to be associated with behavioural disorders and subsequent risks for longer-term mental health problems in children [6, 7]. Increased knowledge of the risk factors associated with ear infection is important in identifying children at risk for recurrent and persistent episodes [8]. 

Reviews of several European countries, the United States, Canada, and Australia have shown risk factors for ear infection to be childcare arrangement, breastfeeding, birth weight, socioeconomic status, and air pollution [9–11]. In a study of a birth cohort in Canada, the strongest risk factors for ear infection were being male, of Aboriginal status, and the child's mother aged less than 20 years [12]. A recent study in Australia has shown poor living conditions, exposure to cigarette smoke, and lack of access to medical care are all major risk factors for ear infection [13]. Ear infections are often mild and frequently resolve themselves within a short period of time. However, the frequency of infection and its associated comorbidity (e.g., fevers and respiratory tract infections) are significant concerns [14]. Substantial co-morbidity has been demonstrated between upper and lower airway infections which include otitis media, tonsillopharyngitis, and lower respiratory infections [15]. 

The prevalence of ear infection, whereby Australian indigenous children had at least an episode in the first year of life, is among the highest in the world and has resulted in a wide range of complications [16, 17]. According to data from Aboriginal Medical Service, rural and remote practitioners manage a greater caring burden than their urban counterparts; hence, affected children have less access to specialist ear health services and longer waiting times [18]. 

Based on a nationally representative sample of Australian children in the same cohort, this study compares the prevalence of ear infection and associated sociodemographic risk factors, health covariates and comorbidity, and health service use at two time points: first at the age of 4 to 5 years and again at the age of 10 to 11 years. This study will provide current insights into evidence-base ear infection in Australian children which in turn may be used in the management, prevention, and intervention of ear infection [19, 20]. 

## 2. Data and Methods 

### 2.1. Longitudinal Study of Australian Children Survey (LSAC)

The LSAC dataset was used to identify the key factors influencing children in the early years of development, using a longitudinal design to examine determinants and life course psychosocial health and well-being outcomes [21, 22]. The study used a two-stage clustered sampling design, stratified by state and by metropolitan/urban status. The Medicare database, which includes 98% of all Australian children, was used for recruitment. Details on development of questions, study design, and nonresponse are reported in other publications [22, 23]. 

Recruitment to the study of over 10,000 children and their families took place from March until November 2004. From 2004, the families have been interviewed every two years. Two child cohorts were recruited in 2004: those born between March 1999 and February 2000 (aged 4-5 year) and those born between March 2003 and February 2004 (aged 0-1 year). Compared to the Australian Bureau of Statistics population estimates, LSAC is broadly representative of the population, with about 10% more educated parents represented and about 5% less non-English speaking families [24, 25]. 

### 2.2. Study Population and Statistical Analyses

The analysis includes 4,983 children aged 4 to 5 years at 2004 baseline and 10 to 11 years in 2010. Sociodemographic characteristics of the sample reported in this study included sex, indigenous status of the study child, and the Australian Bureau of Statistics Socioeconomic Indexes for Areas (SEIFA) [26]. Population sample weights were provided with the dataset to achieve national representation. 

Exposures and outcomes of interest in this study are as follows.

 
*Ear infection* as reported by parent (in most cases this is the child's biological mother) as follows: “Does a study child have ear infection (yes, no)?” It is likely that the reported ear infections include acute otitis media, chronic suppurative otitis media, otitis media with effusion, and uncommon ear diseases. However, nature or severity of ear infections was not reported.
 
*Sociodemographic characteristics* available in this study include sex and indigenous status of the study child, history of being breastfed, school attendance, parental smoking status, and parental school completion. As well, the study reported on respondents' Socioeconomic Indexes for Areas (SEIFA) which ranks the respondent's residential area by the level of social and economic well-being in each region.
 
*Other health conditions* were asked: “Does a study child have any of these ongoing problems… (yes, no)?” Health conditions include asthma, headache, tonsillitis, vision problems, other illnesses, and other infections.
 
*Health service use* was asked “In the last 12 months, have you used any of these services for the study child?… (yes, no)” Health service use includes general practitioners, hospital outpatients, pediatrician, and speech therapy. Other service categories include early intervention services and other specialists.

For variable selection on ear infection and risk factors, health comorbidity, and health service use, we systematically went through the questions available in the LSAC data and included variables of interest for investigation based on relevant national and international literature [9–15]. 

Association between ear infection and risk factors, health co-morbidity, and health service use was reported with the percent prevalence as well as odds ratios and *P* values based on the results from bivariate logistic regression. Data were analysed using Stata 12 [27]. 

## 3. Results 

Prevalence of ear infection was 7.9% (394) at the age of 4 to 5 years and 3.3% (139) at the age of 10 to 11 years.  Table 1 presents sample distribution, ear infection prevalence, and cross-sectional association of ear infection and potential risk factors. The gender ratio within the sample was almost equally split (51% male; 49% female) among the children. Approximately 4% of study children had indigenous status. Approximately 80% reported being breastfed at 1 month, 71% at 3 months, and 57.2% at 6 months. As well, approximately 58.2% of children attended preschool, 24.5% participated in daycare, and 17.4% in kindergarten. Approximately 20% of mothers reported smoking more than once a day; and a similar proportion for fathers. Having less than year 9 school completion or less was reported by 6.9% of mothers and 5.9% of fathers. 

Bivariate association was reported between ear infection and potential risk factors most relevant for children aged 4 to 5 years (Table 1, data not shown for children aged 10 to 11 years). Statistically significant odds ratios and *P* values for risk factors were indigenous status (OR 1.94; *P* = 0.002), not being breastfed at 3 months (OR 1.25; *P* = 0.043), mother smoking more than once a day (OR  1.40; *P* = 0.013), and father smoking at least once a day (OR 1.41; *P* = 0.022). Compared to fathers who had 12+ years of school completion, having fathers who completed year 9 or lower was also a predictor of children with ear infection (OR  1.45; *P* = 0.008). 

Prevalence and cross-sectional bivariate association between ear infection and other health related morbidities were reported in Table 2. At the age of 4 to 5 years, ear infection was statistically associated with asthma (OR 1.55; *P* < 0.001), headache (OR 3.59; *P* = 0.003), other physical disabilities (OR 2.40; *P* = 0.001), other illnesses (OR 1.87; *P* = 0.001), and other infections (OR 3.87; *P* = 0.001). By the age of 10 to 11 year, ear infection was statistically associated with asthma (OR 1.67; *P* = 0.003), headache (OR 2.13; *P* = 0.006), and tonsillitis (OR 4.67; *P* = 0.001). 

Table 3 reports on prevalence and association between ear infection and overall health service usage. At the age of 4 to 5 years, ear infection was strongly and statistically significantly associated with the use of hospital outpatient clinics (OR 2.08; *P* < 0.001) and the use of speech therapy (OR 1.79; *P* < 0.001). By the age of 10 to 11 years, ear infection was found to be strongly and statistically significantly associated with the use of speech therapy (OR 3.24; *P* < 0.001), early intervention service (OR 3.08; *P* = 0.010), general practitioners (OR 2.76; *P* < 0.001), and other specialists (OR 2.12; *P* < 0.001). 

We also applied backward stepwise regression (*P* < 0.05) to determine association between ear infection and overall health service use (data not shown) and found that among children aged 4 to 5 years, ear infection was statistically significantly associated with hospital outpatient clinic usage (OR 2.00; *P* < 0.001) and the use of speech therapy (OR 1.71; *P* < 0.001). Among children aged 10 to 11 years, ear infection was statistically significantly associated with the use of speech therapy (OR 2.70; *P* = 0.003), general practitioners (OR 2.55; *P* < 0.001), and other specialists (OR 1.72; *P* = 0.012). 

## 4. Discussion 

This paper reports on two cross-sectional analyses of Australian children in 2004 (4 to 5 years) and in 2010 (10 to 11 years) of reported ear infection and their associated prevalence with the potential risk factors, health related comorbidity, and overall health service usage. Our prevalence of ear infection was 7.9% among children aged 4 to 5 years and 3.3% among children aged 10 to 11 years. Incidence and prevalence rate of ear infections varied depending on age groups (highest in the first three years); however, differences in definitions and diagnostic measures make comparisons between studies very difficult [16, 18, 28]. For example, a US study of children aged 1–8 years using weekly otoscopy reported prevalence ranging from 7–30% for children aged 5 years and above. Another study was a longitudinal study of Danish children which reported type B tympanogram point prevalence between 11 and 18% at ages 2–5 years and about 7% at ages 6 and 7 years. 

Our study was based on parent-reported data which generally gives lower prevalence than other objective measures; however, our prevalence corresponds to the overall prevalence of ear infection from national data. For example, the National Aboriginal and Torres Strait Islander Health Survey 2004-2005 of the Australian Bureau of Statistics found that among children aged 0–14 years, 4% of indigenous children and 2% of nonindigenous children reported ear infection as a long-term medical condition [29]. In addition, a comprehensive report by Access Economics which analysed Bettering the Evaluation and Care of Health (BEACH) data reported that between 2006 and 2007, 11.5% of children aged less than 15 years had cases of ear infections managed by Australian general practitioners [30]. 

We acknowledge the possible limitation of parent-reported ear infections on behalf of children, including lack of severity reported as well as possible recall bias which may underreport the magnitude of ear infections and which does not take into account repeated ear infection episodes within the same year. However, the use of parent-reported data is not uncommon in international literature. For example, a recent population-based study in Scandinavia has shown self-reported otitis media to be relatively reliable and suggested that any inconsistency in reporting is likely to be associated with less severe episodes [31]. As well, a US study has reported that one of the most common methods to diagnose hearing loss among children was from parental concerns followed by school hearing screens [32]. 

We found various health conditions to be associated with ear infection at the age of 4 to 5 years but only asthma, headache, and tonsillitis were statistically significant at the age of 10 to 11 years. Other studies have also reported upper respiratory tract infections, including otitis media as being comorbid with other symptoms which could further concern the children and their family [14, 15]. However, we note the cross-sectional nature of our study which only allows possible associations to be found, but no causations can be inferred. 

The main strength of this study is the use of the Longitudinal Study of Australian Children which includes a wide array of baseline and repeated questions and its large sample [21, 22]. Our study reported sociodemographic risk factors associated with ear infection to be indigenous status, not being breastfed, parent smoking, and lack of fathers' education. Our findings on risk factors of ear infection are consistent with previous findings elsewhere [8, 9, 11, 33]. A study of 2512 children in North Finland (average age up to 2 years) also confirmed parental smoking, and lack of breastfeeding to be risk factors for ear infection [34]. An Australian longitudinal study of children under the age of 2 years (100 Aboriginal and 180 non-Aboriginal children) showed crowding, attending daycare, and lack of breastfeeding to be strong predictors of otitis media-related bacteria [35]. 

Despite the limitations of the data, we consistently found children with ear infection to be twice as likely to use general practitioners, pediatricians, and other specialists at the age of 10 to 11 years compared to children with no ear infection of the same age group. Our findings on the use of speech therapy and early intervention services show that their use is three times more likely among children with ear infection at the age of 10 and 11 years which could result from referral to the use of specialized services. These results suggest the implications on speech and communication as well as educational development [1, 2]. This study responds to a public health call for evidence on ear infection in Australia [18, 20]. We have provided consistent evidence pointing to the confirmed and identified risk factors, health related comorbidity, and a demand for health and social services required by children affected by ear infection. 

## Figures and Tables

**Table 1:** Prevalence and cross-sectional association of ear infection and potential risk factors.

At baseline age 4 to 5 years	% (*n*)	Ear infection prevalence % (*n*)	Bivariate odds ratio and *P* value
Study child—sex			
Male	50.9 (2537)	8.5 (215)	OR = 1.00
Female	49.1 (2446)	7.3 (179)	OR = 0.85; *P* = 0.131
Study child—indigenous status			
Nonindigenous	96.3 (4794)	7.7 (368)	OR = 1.00
Indigenous	3.8 (187)	13.9 (26)	**OR = 1.94; ** *P * ** = 0.002**
Reported breastfed (months)			
1 month—yes	80.2 (3976)	7.6 (301)	OR = 1.00
1 month—no	19.8 (981)	9.2 (90)	OR = 1.23; *P* = 0.096
3 months—yes	71.0 (3518)	7.4 (260)	OR = 1.00
3 months—no	29.0 (1439)	9.1 (131)	**OR = 1.25; ** *P * ** = 0.043**
6 months—yes	57.2 (2833)	7.3 (207)	OR = 1.00
6 months—no	42.9 (2124)	8.7 (184)	OR = 1.20; *P* = 0.080
Mother—smoking status			
Not smoking	76.9 (3178)	7.4 (235)	OR = 1.00
Less than once/day	3.6 (148)	6.1 (9)	OR = 0.81; *P* = 0.550
More than once/day	19.5 (805)	10.1 (81)	**OR = 1.40; ** *P * ** = 0.013**
Father—smoking status			
Not smoking	75.4 (2451)	7.0 (172)	OR = 1.00
Less than once/day	3.2 (104)	7.7 (8)	OR = 1.10; *P* = 0.792
More than once/day	21.4 (696)	9.6 (67)	**OR = 1.41; ** *P * ** = 0.022**
Mother—school completion			
Year 12+	58.1 (2895)	7.6 (219)	OR = 1.00
Year 10-11	34.9 (1739)	8.2 (143)	OR = 1.10; *P* = 0.376
Year ≤9	6.9 (344)	9.3 (32)	OR = 1.18; *P* = 0.399
Father—school completion			
Year 12+	45.1 (2246)	6.5 (146)	OR = 1.00
Year 10-11	33.8 (1682)	9.1 (153)	**OR = 1.43; ** *P * ** = 0.003**
Year ≤9	5.9 (295)	9.5 (28)	**OR = 1.45; ** *P * ** = 0.008**
SEIFA economic resources			
Equal or above median	54.2 (2699)	7.4 (200)	OR = 1.00
Below median	45.8 (2284)	8.5 (194)	OR = 1.16; *P* = 0.158

**Table 2:** Prevalence and cross-sectional association between ear infection and other health conditions.

Health conditions	Children aged 4 to 5 years			Children aged 10 to 11 years		
	% (*n*)	Ear infection % prevalence (*n*)	Odds ratios and *P* value	% (*n*)	Ear infection % prevalence (*n*)	Odds ratios and *P* value
Asthma						
No		7.2 (283)	OR = 1.00		2.7 (76)	OR = 1.00
Yes	20.6 (1029)	10.7 (110)	**OR = 1.55** *P * ** < 0.001**	33.2 (1381)	4.5 (62)	**OR = 1.67** *P * ** = 0.003**
Headache						
No		7.8 (387)	OR = 1.00		3.1 (123)	OR = 1.00
Yes	0.6 (30)	23.3 (7)	**OR = 3.59** *P * ** = 0.003**	6.0 (248)	6.5 (16)	**OR = 2.13** *P * ** = 0.006**
Tonsillitis						
No					3.0 (123)	OR = 1.00
Yes		N/A		3.0 (125)	12.8 (16)	**OR = 4.67** *P * ** < 0.001**
Vision problems						
No		7.8 (377)	OR = 1.00		3.1 (108)	OR = 1.00
Yes	2.9 (146)	11.6 (17)	OR = 1.56 *P* = 0.092	16.5 (687)	4.5 (31)	OR = 1.47 *P* = 0.063
Other physical disabilities						
No		7.7 (375)	OR = 1.00			
Yes	2.3 (114)	16.7 (19)	**OR = 2.40** *P * ** = 0.001**		N/A	
Other illnesses						
No		7.6 (361)	OR = 1.00		3.2 (128)	OR = 1.00
Yes	5.0 (247)	13.4 (33)	**OR = 1.87** *P * ** = 0.001**	4.8 (198)	5.6 (11)	OR = 1.76 *P* = 0.079
Other infections						
No		7.5 (366)	OR = 1.00		3.3 (138)	OR = 1.00
Yes	2.4 (117)	23.9 (28)	**OR = 3.87** *P * ** < 0.001**	0.6 (27)	3.7 (1)	OR = 1.14 *P* = 0.916

**Table 3:** Prevalence and cross-sectional association between ear infection and health service use.

Health services	Children aged 4 to 5 years			Children aged 10 to 11 years		
	% (*n*)	Ear infection % prevalence (*n*)	Odds ratios and *P* value	% (*n*)	Ear infection % prevalence (*n*)	Odds ratios and *P* value
General practitioners						
No		6.2 (66)	OR = 1.00		1.6 (26)	OR = 1.00
Yes	63.2% (3115)	8.4 (263)	**OR = 1.38**; *P * ** = 0.022**	61.8% (2573)	4.4 (113)	**OR = 2.76**; *P * ** < 0.001**
Hospital outpatients						
No		7.2 (272)	OR = 1.00		3.2 (127)	OR = 1.00
Yes	8.3% (409)	13.9 (57)	**OR = 2.08**; *P * ** < 0.001**	5.6% (231)	5.2 (12)	OR = 1.64; *P* = 0.110
Pediatrician						
No					3.2 (124)	OR = 1.00
Yes		N/A		6.3% (264)	5.7 (15)	**OR = 1.83**; *P * ** = 0.031**
Early intervention services						
No		7.7 (280)	OR = 1.00		3.2 (133)	OR = 1.00
Yes	8.3% (415)	9.1 (49)	OR = 1.20; *P* = 0.260	1.5% (64)	9.4 (6)	**OR = 3.08;** *P * ** = 0.010**
Speech therapy						
No		7.3 (266)	OR = 1.00		3.2 (128)	OR = 1.00
Yes	10.9% (538)	12.3 (63)	**OR = 1.79;** *P * ** < 0.001**	10.4% (513)	9.6 (11)	**OR = 3.24;** *P * ** < 0.001**
Other specialist						
No					3.0 (109)	OR = 1.00
Yes		N/A		11.8% (492)	6.1 (30)	**OR = 2.12;** *P * ** < 0.001**
